# Building a Dementia-Ready Workforce in Malaysia: Family Caregivers’ Insights

**DOI:** 10.1177/14713012261452907

**Published:** 2026-05-21

**Authors:** Eugene Yeoh, Monica Xavier, Cassandra Buffington-Bates

**Affiliations:** 1 161785 Geriatric Unit, Hospital Sungai Buloh, Sungai Buloh, Selangor, Malaysia; 2 School of Pharmacy, 589651 Monash University Malaysia, Subang Jaya, Selangor, Malaysia; 3 279491 Department of Learning Technologies, University of North Texas, Denton, TX, USA

**Keywords:** dementia care, caregiver burden, aged care health service, allied health workforce, health policy

## Abstract

Malaysia’s rapid population aging has intensified the need for dementia care systems that extend beyond hospital-based services. This qualitative study explored the experiences and support needs of family caregivers of persons living with dementia (PLWD) in Malaysia, with the aim of informing dementia workforce development and para-professional training. Semi-structured interviews were conducted with 67 caregivers attending the geriatric unit of a public hospital in Selangor. Responses to open-ended questions on social support, emotional impact, communication with health professionals, economic consequences, and unmet needs were analyzed using structured qualitative content analysis, supported by a human–large language model (LLM) parallel coding approach. Caregivers described substantial emotional burden, including exhaustion, anxiety, social isolation, and feelings of being trapped in a continuous caregiving role. Many reported fragmented information from healthcare professionals, limited awareness of available welfare schemes, and inadequate access to respite, home-based support, and caregiver education. Caregiving also produced major financial strain through lost employment, reduced working hours, depleted savings, and recurring out-of-pocket costs. Across domains, caregivers expressed a strong need for affordable day care, home-based therapy, psychological support, financial assistance, and practical training. These findings indicate that dementia care in Malaysia remains heavily dependent on family caregivers who are expected to absorb unmet system demands with limited formal support. Strengthening the dementia care workforce through structured para-professional roles may help bridge gaps in caregiver education, welfare navigation, post-discharge support, and community-based care. The study underscores the need to align national aging and dementia policies with workforce development strategies that better support both persons living with dementia and the families who care for them.

## Introduction

### Malaysia’s Aging Population and Care Gap

Malaysia’s demographic transition has accelerated in recent decades alongside industrialization, urbanization, and social change. Industrialization gained momentum in the 1970s with the National Economic Plan, which promoted manufacturing and export-oriented growth, increased foreign direct investment, and reshaped the country’s social structure through rising education levels, greater female labor force participation, declining fertility, and longer life expectancy (Department of Statistics Malaysia (DOSM), 2025; Drabble, 2004; Ixora Senior Care, 2025; Mohd et al., 2021; World Bank, nd-a, nd-b). These changes have contributed to a steadily aging population, with DOSM reporting that Malaysians aged 65 years and older now account for 8.0% of the population, up from 7.6% in the previous year, placing the country firmly in the aging-society category (Bernama, 2024; DOSM, 2025). Projections suggest that older adults will make up approximately 15% of the population by 2035, while Malaysia may approach super-aged status by 2040 (Abdullah et al., 2024; Bernama, 2024, 2025).

This demographic shift has created a growing need for health and social care services, especially for chronic disease management, long-term care, and social protection (Azuar, 2025; Nadhirah, 2024; Sum & Mansor, 2025). At the same time, the supply of formal care has not kept pace with demand, particularly as family-based caregiving weakens and younger adults migrate to urban centers for work (Haron et al., 2024; Think CityWorld Bank, 2022; Yue et al., 2024). As a result, care responsibilities continue to fall disproportionately on women in the family, reinforcing gendered patterns of unpaid care work and leaving many households to absorb the burden with limited support (Hamdi & Yahya, 2008; HelpAge International, 2023; Wan Sulaiman, 2019; Yue et al., 2024). These pressures have led to calls for broader policy responses addressing pensions, long-term care financing, gerontological infrastructure, and support for family caregivers (Abdullah et al., 2024; Koshy, 2025).

### Policy Response and Service Reform

In response, the Malaysian government introduced the *Pelan Tindakan Perkhidmatan Kesihatan Warga Emas* (Elderly Healthcare Services Action Plan) 2023–2030, which promotes a person-centered approach to care for older adults (Bernama, 2024; Kementerian Kesihatan Malaysia (KKM), 2023; Ministry of Health Malaysia, 2018). The plan aims to expand geriatric services across primary and tertiary care settings, improve access for rural populations, integrate community-based services, and strengthen healthcare workers’ geriatric knowledge and skills (KKM, 2023). It also emphasizes preventive health, early detection of chronic disease, and rehabilitation to sustain functional independence in later life. Importantly, the action plan recognizes that workforce shortages and competency gaps must be addressed through multi-sectoral collaboration, including training para-professionals to support home-based and transitional care settings (KKM, 2023).

### Chronic Disease and Dementia in Later Life

Aging in Malaysia is increasingly accompanied by a high burden of chronic disease, mirroring trends seen in other countries. Common conditions among older adults include hypertension, diabetes mellitus, hyperlipidemia, ischemic heart disease, stroke, chronic obstructive pulmonary disease, arthritis, chronic kidney disease, cataracts, and depression (Ghazali et al., 2021; Mohd Sidik et al., 2003, 2004, Ministry of Health (MOH), 2024b; Nasaruddin et al., 2025; US National Council on Aging, 2025). These conditions are associated with long-term disability, frequent hospitalization, and cumulative functional decline, all of which place additional pressure on family and health systems (WHO, 2023). They also share modifiable pathways with dementia, including vascular dysfunction, inflammation, and lifestyle-related risk factors, underscoring the need for integrated prevention and care across the life course (Baker et al., 2024; Kementerian Kesihatan Malaysia (KKM), 2023; Livingston et al., 2020, 2024; Ngandu et al., 2015; Sakurai et al., 2025).

Dementia is a growing public health challenge in this context. The global number of people living with dementia continues to rise, with most cases now occurring in low- and middle-income countries (LMICs) (Li et al., 2026; Petersen et al., 2020). Within ASEAN, population aging is expected to intensify sharply over the coming decades, and Malaysia is projected to experience a major decline in its potential support ratio (Wong, 2023). This matters because dementia care in many ASEAN settings remains heavily reliant on families, with limited publicly funded long-term care and a comparatively small formal care workforce (Giebel et al., 2025; Li et al., 2026; Mattap et al., 2022; Vo & Fong, 2025). In practice, this leaves many caregivers managing complex and progressive illness trajectories at home with little training or respite. Dementia, therefore, cannot be understood only as a biomedical condition; it is also a social and systems-level challenge that exposes weaknesses in care infrastructure, labor policy, and family support.

Within Malaysia, dementia prevalence is increasing among older adults and is shaped by age, sex, education, ethnicity, and living arrangement. A cross-sectional study in Muar, Johor, reported dementia prevalence of 13.4% among adults aged 60 years and above, rising steeply with age and reaching 28.1% among those aged 75 years and older (Tajudin et al., 2016). Higher prevalence was also seen among women, people with no formal education, widowed or divorced persons, and nursing home residents (Tajudin et al., 2016). These findings highlight the need for dementia strategies that address both biomedical risk and broader social determinants.

### Caregiver Burden and Workforce Shortages

The burden of dementia extends well beyond the person living with dementia. As the disease progresses, increasing assistance with activities of daily living is required, placing emotional, physical, and financial demands on family caregivers (BrightFocus Foundation, nd; Prizer & Zimmerman, 2018). In Malaysia, caregivers often manage these responsibilities with limited structured support or professional guidance (MOH, 2024a). This reflects a broader global problem: more than 75% of people with dementia worldwide remain undiagnosed or without coordinated care, particularly in LMICs (Alzheimer’s Disease International, 2021). Recent international reviews also show that direct care workers are essential to dementia care but remain understudied, especially regarding recruitment, retention, training pathways, and the quality of person-centered support (Soh et al., 2025; Travers Altizer et al., 2025). Evidence from Asian and LMIC long-term care settings further indicates that shortages, low wages, limited training, and weak support structures undermine the delivery of effective dementia care (Guan et al., 2025; Li et al., 2026).

The complexity of dementia care requires a multidisciplinary, person-centered approach that integrates medical, psychosocial, and functional support (ATrain Education, nd; Grand et al., 2011; Saragosa et al., 2024; Warren, 2023; Zucchella et al., 2018). However, Malaysia continues to face a shortage of health professionals trained in geriatrics and dementia care, resulting in gaps in continuity of care, coordination, and caregiver education (MOH, 2024a; WHO, 2023). This shortage reinforces the need for a structured para-professional workforce that can bridge clinic-based services and home-based caregiving, while also supporting caregiver navigation, education, and psychosocial assistance (KKM, 2023).

### Theoretical Framework

This study is informed by Pearlin’s Stress Process Model of caregiving, which conceptualizes caregiver outcomes as shaped by background characteristics, primary stressors, secondary role strains, and available coping resources and social support (Aneshensel and Avison 2025; Hilgeman et al., 2009; Judge et al., 2010; Mausbach et al., 2012; Pearlin et al., 1990; Shah et al., 2010; Zhao et al., 2022). In this framework, caregiving burden is not simply a function of the care recipient’s condition but is also produced by structural and social conditions that shape how stress accumulates and how coping resources are distributed. In the Malaysian context, these processes are further shaped by gendered care norms and limited formal support, making the stress process especially relevant for interpreting caregiver experience. This lens helps explain why caregiving in our study was associated not only with emotional strain, but also with work disruption, financial depletion, and unmet informational needs.

### Study Objectives

This article aims to examine the experiences and support needs of family caregivers of persons living with dementia in Malaysia, and to identify how current occupational structures and service systems fall short of these needs. By linking caregiver experience to stress-process theory and to national workforce planning, the study also seeks to inform the development of para-professional and allied health roles that are better aligned with dementia care needs. Specifically, we explore the implications of Malaysia’s rapid population aging, the burden of chronic disease and dementia, and the extent to which current training and occupational standards are equipped to support sustainable, high-quality care. We also consider how national policy instruments such as the *Pelan Tindakan Perkhidmatan Kesihatan Warga Emas 2023–2030* and the *Dementia Action Plan 2023–2030* may be operationalized through workforce development, service integration, and caregiver support (KKM, 2023; MOH, 2024a).

## Methods

### Materials and Study Setting

This study was conducted in the geriatric unit at Hospital Sungai Buloh, Selangor, Malaysia, and explored the experiences of informal and formal caregivers of older adults with dementia receiving care at this facility. Direct interviews with persons living with dementia (PLWD) were not undertaken due to concerns about their decisional capacity and the feasibility of conducting in-depth qualitative interviews (Beuscher & Grando, 2009; Conway et al., 2023; Runacres & Herron, 2023). The research design centered on caregiver perspectives, consistent with recommendations to prioritize collateral informants for moderate-to-severe cognitive impairment.

Caregivers completed a semi-structured interview that included a subset of open-ended questions (Q1–Q5f) exploring sources of support, emotional impact of caregiving, communication with health professionals, perceived financial and occupational consequences, and views on existing services and unmet needs. The interview guide was developed using best-practice qualitative approaches with dementia caregivers, emphasizing open-ended prompts to elicit rich descriptions of lived experience, burden, and perceptions of formal and informal support systems (Beuscher & Grando, 2009; Runacres & Herron, 2023). The research was also guided by the COREQ checklist to enhance transparency in sampling, data collection, and analysis. Consistent with the study’s interpretive orientation, the methods were designed to capture caregiver burden not only as an individual experience but also as a reflection of broader service and workforce constraints, in line with the theoretical framing described in the Introduction and further discussed in the Discussion.

### Participants

The target population was 100 primary caregivers of PLWD who attended the geriatric clinic or were admitted to the geriatric unit for assessment or follow-up. A consecutive convenience sampling approach was used, whereby all eligible caregivers attending clinic sessions during the data collection period were approached in the waiting area and invited to participate. There were no refusals, as all eligible caregivers approached agreed to participate. Basic sociodemographic information was collected for caregivers (age, gender, relationship to PLWD, co-residence, caregiving duration and intensity, employment status, and selected socioeconomic indicators) and for PLWD (age at diagnosis, gender, duration of illness, and previous occupation).

An interim cutoff was applied and analyzed to present preliminary results at the Malaysian Congress of Geriatric Medicine. The cutoff date was 23 May 2025, and 67 participants were included, all of whom were family caregivers who had provided hands-on care for at least one year and were involved in day-to-day decision-making. During iterative coding, no new substantive codes emerged in the final interviews, and thematic redundancy was observed across key domains (social support, emotional impact, information, and economic consequences), suggesting that data saturation had been reached for the study’s aims. Inclusion criteria required caregivers to be aged 18 years or older, to be able to communicate in Malay, English, or Tamil, and to be willing to provide written informed consent.

### Data Collection Procedures

Data collection was conducted by two researchers with clinical and research experience relevant to geriatric and dementia care, and took place in the geriatric clinic while caregivers and PLWD were waiting for their appointments with the geriatrician. Depending on clinic flow and caregiver availability, individual interviews ranged from approximately 30 minutes to 2 hours and were conducted in a private and quiet area to protect privacy. No repeat interviews were conducted; each participant was interviewed once in a single session. No audio recording was used; interviewers recorded verbatim responses on standardized forms, which were later compiled into a unified dataset for coding.

For the present analysis, the focus is on six core open-ended questions: Q1 (sources and adequacy of social and service support), Q2 (emotional impact and coping), Q3 (information and communication with healthcare professionals), Q4 (impact on personal life and mental/physical health), Q5a–d (economic, employment and financial consequences of caregiving), and Q5e–f (awareness, use and perceived gaps in assistance schemes and services). When caregivers became visibly distressed, interviews were paused, and support was offered in accordance with the study’s ethical procedures and the clinical setting.

### Data Analysis Approach

Coding and data management were performed using spreadsheet files. Given the qualitative nature of Q1–Q5f, the analysis combined a phenomenological thematic interpretation (Daly-Lynn et al., 2022; Li et al., 2024; Tambunan & Simbolon, 2023; Wong et al., 2025) with a structured content analysis using a prespecified codebook. Participant checking was not performed.

First, all free-text responses were collated and de-identified. Two parallel code sets were generated: (1) human expert codes (Human_codes_full.csv) developed iteratively by an experienced qualitative researcher in dementia and caregiving, and (2) machine-assigned codes (AI_codes_full.csv) produced by a large language model (LLM) fine-tuned to apply the same code labels. The codebook captured domains including social support (e.g., SS_FAM_INF, SS_NO_SUPPORT, SS_UNMET_NEEDS), emotional impact and coping (e.g., EMO_EXHAUSTION_STRAIN, EMO_DEPRESSION_ANXIETY, COPING_RELIGIOUS_SPIRITUAL), information and communication (e.g., INFO_POOR_UNINFORMED, INFO_PARTIAL, COMM_GOOD_PROF_RELATION, COMM_DIFFICULT_BARRIERS), financial and occupational impact (e.g., EB_EMPLOYMENT_LOSS, EB_REDUCED_INCOME_HOURS, EB_DEBT_SAVINGS_RETIREMENT), and explicit requests or unmet needs for services and assistance (e.g., REQ_FORMAL_SERVICES, REQ_FINANCIAL, REQ_PSYCHOSOCIAL_SUPPORT, REQ_INFO_EDUCATION).

In the present paper, descriptive content analysis of the human-coded dataset was used to quantify the proportion of caregivers whose narratives contained at least one code in each domain (e.g., emotional strain, economic impact, unmet service need). Frequencies of specific request codes in Q5e–Q5f were examined to characterize the types of services and supports caregivers felt were missing or inadequate. AI-generated codes were used secondarily to examine concordance and confirm that observed thematic patterns (e.g., high emotional burden, financial strain, unmet formal services) were robust across coder types. To assess the robustness of themes, the machine-assigned codes were compared with human expert codes in a subset of responses. Concordance for the presence/absence of major domains (emotional burden, financial strain, unmet service needs, and information/communication issues) was high, with most discrepancies reflecting differences in granularity rather than direction (e.g., the LLM grouping multiple nuanced emotional descriptors under a broader “stress” label). The LLM showed limitations in capturing culturally specific expressions and sarcasm, so human interpretation was retained as the primary analytic reference. Given these findings, the present paper reports descriptive analyses based on human coding, with AI codes used secondarily as a validity check to confirm that the core thematic patterns were not artefacts of a single coder.

As the analysis progressed, recurring patterns of emotional exhaustion, role strain, economic depletion, and unmet service needs emerged across interviews, reinforcing the interpretation that the data were sufficiently rich to support the study’s aims. This approach allowed the study to retain both thematic depth and structured comparability across the principal domains of caregiver experience.

### Ethical Considerations

Ethical approval for the overall caregiver study was obtained from the institutional review board and the Malaysian Medical Research and Ethics Committee. All participants provided written informed consent after receiving a clear explanation of the study aims, voluntary participation, and the right to withdraw without affecting clinical care. Interviews were conducted sensitively, with interviewers monitoring for signs of distress and pausing or redirecting the conversation if needed; information on available psychosocial support services was offered when high distress or unmet mental health needs were disclosed. Data were anonymized at the point of transcription, with unique reference IDs used in the analytic datasets to protect participant identity. These procedures were intended to minimize participant burden while preserving the authenticity of caregiver accounts in a busy clinical setting.

## Results

### Caregiver Sample

The 67 caregivers were predominantly female (80.7%), and were most commonly adult children (59.7%) or spouses (34.4%). Among the 62 respondents who provided caregiving duration data, the median duration was 4 years (IQR 2.5–6.0). Although the inclusion criteria specified a minimum of one year of caregiving experience, three caregivers with less than one year of dementia caregiving were retained in the analysis because they had been co-residing with and caring for PLWD prior to diagnosis, either due to other medical needs or pre-existing caregiving arrangements. One respondent reported caregiving for two parents with differing durations; these were analyzed as separate observations. The mean age of caregivers was 56.8 ± 13.3 years, with a range of 31–81 years. Table 1 summarizes PLWD characteristics, and Table 2 summarizes caregiver characteristics. This sample profile reflects the gendered and family-centered organization of dementia care in the Malaysian context, in which caregiving responsibility is concentrated among women and adult children.

**Table 1.** Demographics of People Living With Dementia (PLWD)

Category	Sub-category	Frequency	Percentage
Gender	Female	30	44.8
	Male	37	55.2
Age of diagnosis	45–54	1	1.5
	55–64	9	13.4
	65–74	31	46.3
	75–84	17	25.4
	85–94	5	7.5
	Undisclosed	4	6.0
Ethnicity	Chinese	34	50.7
	Malay	22	32.8
	Indian	11	16.4
Number of caregivers	1	24	35.8
	2	25	37.3
	3	13	19.4
	4	2	3.0
	5	2	3.0
	10	1	1.5
Range of age of PLwD	​	54–91	​
Mean age of PLwD	​	71.4 ± 8.4	​

**Table 2.** Demographics of Caregivers

Category	Sub-category	Frequency	Percentage
Gender	Female (daughter)	31	46.3
	Female (DIL)	1	1.5
	Female (employee)	2	3.0
	Female (wife)	19	28.4
	Female (undisclosed)	1	1.5
	Male (husband)	4	6.0
	Male (son)	9	13.4
Age	30–39	7	10.4
	40–49	15	22.4
	50–59	15	22.4
	60–69	15	22.4
	70–79	11	16.4
	80–89	1	1.5
	Undisclosed	3	4.5
Range of age of caregiver	​	31–81	​
Mean age of caregiver	​	56.8 ± 13.3	​
Caregiving duration (years)	<1	3	4.8
	1–3	23	36.5
	>3–5	15	23.8
	>5–8	14	22.2
	>8–10	2	3.2
	>10	6	9.5
Range of duration (years)	​	Q1: 2.5	​
		Q3: 6.0	
Median duration (years)	​	4	​

A total of 67 caregivers contributed responses to Q1–Q5f that were suitable for coding, predominantly female spouses or daughters providing long-term care for 1 to over 20 years. All respondents were family caregivers. The results of the caregivers’ responses are presented in Table 3, and a description of all codes is provided in Table 4. Across the dataset, the core pattern was intensive, sustained caregiving within households with limited access to structured formal support.

**Table 3.** Caregivers’ Coded Responses to Q1–Q5f in Key Themes Aligned With Para-Professional Training and Service Gaps

Theme	Main codes	Typical caregiver experiences	Implications for para-professional training and deployment
Social and service support (Q1)	SSFAMINF, SSFRIENDCOMM, SSNOSUPPORT, SSFAMLIMITED, SSUNMETNEEDS, SSFORMALHEALTH, SSFORMALGOVWELFARE, SSRESPITEDAYCARE	Most caregivers rely heavily on spouses, children, and extended family, with some support from friends, neighbors, and faith communities. A substantial subgroup reports “no help” or very limited family support and struggles to access respite, day care, or home-based services despite recognizing them as helpful when available.	Indicates the need for trained community-based para-professionals and allied health workers (e.g., care aides, community nurses, therapists) to provide structured respite, home visits, and support with navigating welfare schemes, rather than relying almost exclusively on informal family networks.
Emotional and mental health impact (Q2, Q4)	EMOEXHAUSTIONSTRAIN, EMODEPRESSIONANXIETY, EMOANGERIRRITABILITYGUILT, PLMENTALHEALTHIMPACT, PLTIMELOSSSOCIALISOL, PLROLERESTRICTION, PLACCEPTANCEADJUSTMENT, EMOPOSITIVEMEANING, COPINGEMOTIONFOCUSEDDISTRACT, COPINGRELIGIOUSSPIRITUAL, COPINGNONENOTIME	Narratives frequently describe being “tired,” “trapped,” “hopeless,” lonely and socially isolated, with some reporting depressive symptoms and anger or guilt towards the person with dementia. Coping is often emotion-focused (distraction, withdrawal, prayer), and a notable subset reports having “no time” or “no strategy” to cope, despite severe strain.	Highlights the absence of accessible, dementia-informed psychological and psychosocial support for caregivers and underscores the need to train para-professionals in basic mental health screening, supportive counselling, stress-management, and referral pathways for caregiver distress.
Information and communication with health professionals (Q3)	INFOWELLINFORMED, INFOPOORUNINFORMED, INFOPARTIAL, INFOSEEKINGSELFLEARN, COMMDIFFICULTBARRIERS, COMMGOODPROFRELATION, COMMLANGUAGECULTURE	Some caregivers feel well informed and report good relationships with geriatricians, especially when clinicians explain disease progression and involve them in decisions.​ Many others feel only partly or poorly informed, describe vague or inconsistent messages, communication barriers, and the need to “read on my own” or rely on peers for dementia information	Indicates that dementia-specific communication and education competencies are not yet consistently embedded across front-line staff; training for nurses, allied health, and community workers should include structured caregiver education, culturally sensitive communication, and ongoing information support.
Employment and economic impact (Q5a–d)	EBEMPLOYMENTLOSS, EBREDUCEDINCOMEHOURS, EBCAREERPROGRESSLIMIT, EBDEBTSAVINGSRETIREMENT, EBDIRECTMEDICAL, EBFORMALCARESERVICES, EBSUPPLIESDIAPERSNUTRITION, EBOTHERDIRECTCOSTS, EBLIFESTYLECHANGE, EBOVERALLIMPACTSEVERE, EBOVERALLIMPACTMILDNONE, EBEXISTINGASSISTANCE	Many caregivers report quitting work, reducing hours, or sacrificing career progression to provide care, leading to reduced income and long-term financial insecurity. Household budgets are strained by recurrent costs for medical care, paid carers, diapers, nutritional supplements, equipment, and home modifications; some families benefit from pensions or subsidies but still describe the overall impact as “severe.”	Demonstrates that dementia caregiving carries a substantial economic burden, suggesting para-professionals (e.g., medical social workers, community case managers) should be trained to assess financial strain, advise on benefits, coordinate cost-effective services, and advocate for caregiver-friendly workplace policies.
Awareness and use of assistance schemes (Q5e)	ASSTKNOWNACCESSED, ASSTUNAWARE, EBUNMETFINANCIALNEEDS, EBEXISTINGASSISTANCE	Some caregivers know and use schemes such as JKM allowances, pensions, or OKU benefits, but others are completely unaware of available assistance or uncertain about how to apply. Even among users, many emphasize that current assistance only partially offsets the high costs of dementia care and that important needs (e.g., equipment, ongoing supplies) remain uncovered.	Points to a systemic gap in benefit navigation; para-professionals and allied health staff require training in social welfare literacy and care coordination so they can proactively inform caregivers, assist with applications, and ensure better linkage between clinical services and welfare systems.
Explicit requests and unmet service needs (Q5f)	REQFORMALSERVICES, REQFINANCIAL, REQPSYCHOSOCIALSUPPORT, REQINFOEDUCATION, REQPOLICYFLEXIWORK, REQNONEORUNSPECIFIED	Caregivers explicitly request affordable or subsidized day-care, respite, home-based therapy, professional home carers, nursing home subsidies, and equipment support.​ Many also call for financial assistance, tax relief, emotional and psychological support, structured dementia education, and flexible work policies that recognize caregiving responsibilities.	Provides direct evidence that current training and deployment of geriatric and dementia-focused para-professionals and allied health staff do not meet caregivers’ evolving needs; supports arguments for workforce expansion, dementia-specific competency frameworks, and integration of caregiver support, education, and advocacy into routine practice.

**Table 4.** Codes Used in Key Thematic Analysis

Domain	Code label	Description
Assistance available	ASST_INADEQUATE	Assistance inadequate
Assistance available	ASST_KNOWN_ACCESSED	Knows and uses assistance
Assistance available	ASST_KNOWN_NOT_ACCESSED	Knows but not using assistance
Assistance available	ASST_UNAWARE	Unaware of assistance
Information communication	COMM_DIFFICULT_BARRIERS	Difficulties/barriers in communication
Information communication	COMM_GOOD_PROF_RELATION	Good communication with professionals
Information communication	COMM_LANGUAGE_CULTURE	Language/cultural mismatch
Emotional impact	COPING_EMOTION_FOCUSED_DISTRACT	Emotion-focused/distraction coping
Emotional impact	COPING_NONE_NO_TIME	No coping strategy/no time
Emotional impact	COPING_PROBLEM_FOCUSED	Problem-focused coping
Emotional impact	COPING_RELIGIOUS_SPIRITUAL	Religious/spiritual coping
Economic burden	EB_CAREER_PROGRESS_LIMIT	Limited career advancement
Economic burden	EB_DEBT_SAVINGS_RETIREMENT	Debt, savings, and retirement funds
Economic burden	EB_DIRECT_MEDICAL	Direct medical and care costs
Economic burden	EB_EMPLOYMENT_LOSS	Job loss or leaving work
Economic burden	EB_EXISTING_ASSISTANCE	Existing financial assistance
Economic burden	EB_FORMAL_CARE_SERVICES	Paid carers, daycare, and nursing home costs
Economic burden	EB_LIFESTYLE_CHANGE	Lifestyle change/reduced budget
Economic burden	EB_OTHER_DIRECT_COSTS	Other direct caregiving costs
Economic burden	EB_OVERALL_IMPACT_MILD_NONE	Mild or minimal financial impact
Economic burden	EB_OVERALL_IMPACT_SEVERE	Severe overall financial impact
Economic burden	EB_REDUCED_INCOME_HOURS	Reduced hours/income
Economic burden	EB_SUPPLIES_DIAPERS_NUTRITION	Diapers, nutritional and other consumable supplies
Economic burden	EB_UNMET_FINANCIAL_NEEDS	Unmet financial support needs
Emotional impact	EMO_ANGER_IRRITABILITY_GUILT	Emotional exhaustion/chronic strain
Emotional impact	EMO_DEPRESSION_ANXIETY	Emotional exhaustion/chronic strain
Emotional impact	EMO_EXHAUSTION_STRAIN	Emotional exhaustion/chronic strain
Emotional impact	EMO_POSITIVE_MEANING	Positive emotions and meaning-making
Information communication	INFO_PARTIAL	Partial/mixed understanding
Information communication	INFO_POOR_UNINFORMED	Poorly informed/uninformed
Information communication	INFO_SEEKING_SELF_LEARN	Self-directed information seeking
Information communication	INFO_WELL_INFORMED	Well-informed about dementia
Personal life impact	PL_ACCEPTANCE_ADJUSTMENT	Acceptance and adaptation
Personal life impact	PL_MENTAL_HEALTH_IMPACT	Mental health impact
Personal life impact	PL_MINIMAL_IMPACT	Minimal or manageable impact
Personal life impact	PL_PHYSICAL_HEALTH_IMPACT	Physical health impact
Personal life impact	PL_ROLE_RESTRICTION	Role restriction/identity change
Personal life impact	PL_TIME_LOSS_SOCIAL_ISOL	Loss of personal time/social isolation
Support requests	REQ_FINANCIAL	Requests for financial support
Support requests	REQ_FORMAL_SERVICES	Requests for formal services
Support requests	REQ_INFO_EDUCATION	Requests for information/education
Support requests	REQ_NONE_OR_UNSPECIFIED	No or unspecified requests
Support requests	REQ_POLICY_FLEXI_WORK	Policy/flexible work requests
Support requests	REQ_PSYCHOSOCIAL_SUPPORT	Requests for psychosocial/emotional support
Social support	SS_FAM_INF	Strong informal family support
Social support	SS_FAM_LIMITED	Limited or strained family support
Social support	SS_FORMAL_GOV_WELFARE	Government/NGO financial or practical aid
Social support	SS_FORMAL_HEALTH	Formal healthcare services
Social support	SS_FRIEND_COMM	Friends/community/faith support
Social support	SS_NO_SUPPORT	No or virtually no support
Social support	SS_RESPITE_DAYCARE	Respite care/daycare
Social support	SS_UNMET_NEEDS	No or virtually no support

### Social Support and Service Use (Q1)

Caregivers most frequently reported receiving day-to-day support from immediate and extended family members (SS_FAM_INF) and, to a lesser extent, friends, neighbors, and faith communities (SS_FRIEND_COMM). At the same time, a substantial subset explicitly reported having “no support” or inadequate help (SS_NO_SUPPORT, SS_FAM_LIMITED, SS_UNMET_NEEDS), highlighting a heavy reliance on a single primary caregiver even when extended family was present. Formal services mentioned as particularly helpful included the Hospital Sungai Buloh geriatric team and Cognitive Stimulation Therapy (SS_FORMAL_HEALTH), as well as government welfare schemes such as JKM or SOCSO (SS_FORMAL_GOV_WELFARE). Finally, caregivers highlighted the benefit of respite and day care services, home-based therapy, and professional carers (SS_RESPITE_DAYCARE, EB_FORMAL_CARE_SERVICES), underscoring the value of structured para-professional input when such resources are available. In several accounts, support was described as present in principle but inconsistent in practice, suggesting that care networks often depended on informal goodwill rather than reliable service pathways.

### Emotional Impact and Coping (Q2, Q4)

Across Q2 and Q4, the majority of caregivers described a high emotional burden, commonly coded as EMO_EXHAUSTION_STRAIN and EMO_DEPRESSION_ANXIETY, with narratives of feeling “tired,” “trapped,” “hopeless,” or in “survival mode.” Many linked caregiving to loneliness, loss of social life, and pervasive stress, captured by PL_TIME_LOSS_SOCIAL_ISOL, PL_MENTAL_HEALTH_IMPACT, and PL_ROLE_RESTRICTION, whereas a smaller group expressed primarily positive meaning or acceptance (EMO_POSITIVE_MEANING, PL_ACCEPTANCE_ADJUSTMENT). Coping strategies included emotion-focused approaches such as distraction, entertainment, or “shutting down” (COPING_EMOTION_FOCUSED_DISTRACT), religious or spiritual practices (COPING_RELIGIOUS_SPIRITUAL), and, less commonly, problem-focused strategies such as developing routines or seeking training. Finally, a notable subset of caregivers reported having “no time” or “no strategy” to cope, often leaving them in a state of continuous burnout (COPING_NONE_NO_TIME). Verbatim narratives reinforced the intensity of this strain, including descriptions such as “Emotionally tired, depressed, no life of my own” and “Angry and hopeless. No coping strategy, as there is no time to manage my own stress.”

A smaller but important set of narratives also pointed to intrafamilial conflict and identity strain, with caregivers describing accusations from relatives, marital tension, and feelings of being trapped in an all-encompassing role. These accounts illustrate that caregiver distress was not only emotional but also relational, affecting family dynamics and self-concept.

### Information and Communication With Healthcare Professionals (Q3)

Caregivers’ experiences of information provision and communication were heterogeneous. A proportion felt “well informed” about dementia and its progression and reported good relationships with healthcare professionals (INFO_WELL_INFORMED, COMM_GOOD_PROF_RELATION), often citing specific geriatricians or teams, such as those at Hospital Sungai Buloh, who explained the illness clearly and were responsive via clinic visits or messaging. In contrast, many others described being only partly informed or poorly informed (INFO_PARTIAL, INFO_POOR_UNINFORMED), reporting that the information was vague, overly technical, or not volunteered unless they repeatedly asked. Communication barriers were common, including perceptions that staff did not fully understand caregiver struggles, time pressure, language mismatches, or experiences of being ignored or coerced into administrative tasks (COMM_DIFFICULT_BARRIERS, COMM_LANGUAGE_CULTURE), pointing to gaps in dementia-specific communication skills and caregiver education among front-line staff. Narratives highlighted specific frustrations, such as staff at emergency departments not recognizing dementia-related restlessness or nurses insisting on survey participation despite a caregiver’s urgent schedule. These findings underscore that while some caregivers experience effective guidance, a significant portion must rely on self-directed learning to compensate for inadequate professional communication. The result is a fragmented informational environment in which caregivers are left to interpret disease progression, anticipate behavioral changes, and navigate services largely on their own.

### Impact on Employment and Household Economy (Q5a–d)

Economic and work-related consequences of caregiving were prominent throughout the study, with narratives illustrating how the demands of intensive care served as a catalyst for financial and occupational instability. Numerous caregivers reported quitting jobs to provide full-time care, reducing their working hours, or stagnating in their careers, themes captured by the codes EB_EMPLOYMENT_LOSS, EB_REDUCED_INCOME_HOURS, and EB_CAREER_PROGRESS_LIMIT. For example, one caregiver described having to resign specifically to focus on her mother’s intensive care needs, while others noted that employer dissatisfaction with frequent leave for medical appointments forced a transition to part-time work. The narratives also revealed serious financial strain, most notably the depletion of personal savings and erosion of retirement funds (EB_DEBT_SAVINGS_RETIREMENT), which emerged as the most frequent economic theme, affecting 40 respondents. This long-term insecurity was compounded by immediate, recurring out-of-pocket expenses for medical care (EB_DIRECT_MEDICAL), paid professional carers (EB_FORMAL_CARE_SERVICES), and consumables such as diapers and nutritional supplements (EB_SUPPLIES_DIAPERS_NUTRITION). Verbatim accounts highlighted that costs for basic supplies like diapers could reach RM700 per month, placing a significant burden on those with fixed incomes. To manage these costs, many families were forced into significant lifestyle changes (EB_LIFESTYLE_CHANGE), including becoming more thrifty and tightening household budgets. While some families were able to mitigate these impacts through existing assistance (EB_EXISTING_ASSISTANCE), such as government pensions or welfare subsidies like JKM, the overall impact was still frequently characterized as EB_OVERALL_IMPACT_SEVERE. These findings show how caregiving creates cumulative financial pressure that extends beyond immediate care expenses to long-term threats to household security, retirement planning, and employment continuity.

### Awareness and Use of Assistance Schemes (Q5e)

Responses to Q5e revealed a significant gap between the availability of formal aid and its actual utilization, characterized by limited awareness and uptake of caregiving support schemes. Caregivers either reported knowing and accessing at least one scheme—such as JKM cash transfers, SOCSO, OKU registration, or Skim Tunai Ramah (ASST_KNOWN_ACCESSED)—or, significantly more frequently, reported being entirely unaware of any assistance or the application processes (ASST_UNAWARE). Quantitative analysis supports this disparity, with 19 respondents explicitly coded as unaware of assistance compared to only 7 who were currently accessing supports. Even among the minority with some support, narratives frequently highlighted partial coverage and a failure of formal aid to keep pace with the cumulative costs of care. For example, one caregiver noted that the government subsidized only “one side of [a] hearing aid,” while others reported that modest cash assistance was insufficient to cover recurring costs, such as RM700 per month for diapers. These persistent unmet financial needs (EB_UNMET_FINANCIAL_NEEDS) suggest a critical lack of navigation support and limited integration between clinical teams and welfare services. In practical terms, many caregivers appeared to know that support existed but not how to access it, indicating that the barrier was not only resource scarcity but also weak care coordination.

### Explicit Service and Training Needs (Q5f)

Q5f responses provided a clear picture of what caregivers perceived as missing from the current system. Commonly requested supports included affordable or subsidized day care and respite services, home-based physiotherapy and occupational therapy, professional home carers or domestic helpers at reasonable cost, and nursing home subsidies (REQ_FORMAL_SERVICES, SS_RESPITE_DAYCARE, EB_FORMAL_CARE_SERVICES). Many caregivers also explicitly requested financial assistance, tax relief, or targeted subsidies for diapers, nutritional supplements, and other recurring expenses (REQ_FINANCIAL), as well as emotional and psychological support for caregivers (REQ_PSYCHOSOCIAL_SUPPORT). Several responses called for more structured caregiver education, dementia-specific information, and public or workplace policies, such as flexible work arrangements for caregivers (REQ_INFO_EDUCATION, REQ_POLICY_FLEXI_WORK), thereby highlighting gaps in the training and deployment of para-professional and allied health staff capable of delivering caregiver education, counseling, and coordinated community-based support. Overall, the requests pointed to a system that is responsive in isolated pockets but not yet organized around the sustained practical and psychosocial needs of family caregivers.

Taken together, the results show a consistent pattern across domains: caregivers experienced strong informal family support when available, but also substantial emotional exhaustion, weak communication with professionals, financial strain, and low awareness of formal assistance. These findings provide the empirical basis for the workforce and policy recommendations developed in the Discussion.

## Discussion

The caregiving context identified in this study reflects patterns consistent with stress-process frameworks, in which intensive care demands and limited formal support contribute to compounded emotional distress and economic strain (Aneshensel and Avison, 2025; Hilgeman et al., 2009; Judge et al., 2010; Mausbach et al., 2012; Pearlin et al., 1990; Shah et al., 2010; Zhao et al., 2022). Within the Malaysian socio-cultural context, these pressures are further shaped by gendered expectations that position women as default caregivers (Hilgeman et al., 2009; Shah et al., 2010; Zhao et al., 2022). The predominance of female spouses and daughters in our sample is therefore not merely descriptive; it reflects a broader social organization of care that amplifies vulnerability to burden, social isolation, and employment disruption.

Overall, caregivers described a pattern of sustained emotional, physical, and financial strain. Despite regular contact with geriatric services, many reported fragmented information, limited practical guidance, and communication barriers with health professionals. As a result, caregivers frequently relied on self-directed learning rather than structured education from trained staff. Across community, respite, and home-based services, support was often described as patchy, inaccessible, or poorly tailored to dementia care. Caregivers consistently identified unmet needs for affordable daycare, respite care, home-based therapy, psychological support, financial assistance, and practical caregiving training (Yaw et al., 2024).

These findings suggest that current geriatric and dementia care pathways in Malaysia do not yet sufficiently integrate para-professional and allied health workers into community and home-based support. The unmet needs described by caregivers’ map directly onto a para-professional workforce model: affordable daycare and respite services point to dementia aides and activity coordinators; requests for home-based therapy and caregiving guidance suggest roles in basic rehabilitation support and caregiver education; and limited awareness of assistance schemes highlights the need for care navigators who can connect families to welfare benefits and community resources. In this sense, family caregivers are compensating for systemic gaps that a more structured care workforce should address.

Our findings also align with broader dementia workforce literature showing that direct care workers are essential to dementia support yet remain underexamined, particularly regarding recruitment, retention, and training pathways (Travers Altizer et al., 2025). In the Malaysian context, the absence of a robust para-professional tier shifts this work to unpaid family caregivers, who provide complex care without formal preparation, compensation, or respite. This creates a hidden workforce of unpaid labor that absorbs the practical and emotional costs of dementia care.

The Ministry of Health has already taken important steps through national policy, but these initiatives require a more defined workforce structure to be fully operationalized. The National Occupational Skills Standard (NOSS) for health services should therefore be updated to include para-professional levels 1–3, providing a competency-based framework for universities, colleges, and TVET institutions (Council of Accredited Social Work Educators, 2018; Department of Skills Development, n.d.). In practical terms, this would create a formal pathway for dementia aides, care coordinators, and community support workers whose roles align closely with the gaps identified in this study. Such revision would strengthen workforce capacity, better align training with national policy goals, and respond more directly to caregivers’ reports of limited knowledge, low confidence, and difficult transitions from hospital to home (Bosman et al., 2025; Cotton et al., 2021; D’Silva, 2025; Goodson et al., 2021; Institute of Public Health, 2019; Lee et al., 2025; Thai et al., 2024). It would also support the move toward internationally recognized dementia care standards (Ashbourne et al., 2021; Surr et al., 2017; Weiss et al., 2020).

Translating these recommendations into practice will require attention to fiscal constraints, regulatory approval for new occupational categories, and possible concerns about role boundaries among existing professional groups. Close collaboration between the Ministry of Health, Ministry of Human Resources, training institutions, and professional councils will be essential to ensure that para-professional roles complement rather than compete with existing disciplines and remain sustainable over time. These implementation considerations are important because workforce reform depends not only on training design, but also on governance, supervision, and financing (Tran et al., 2025).

A para-professional workforce could also strengthen continuity of care across the hospital-to-home transition. These workers could support medication adherence, appointment scheduling, basic counseling, welfare navigation, and family mediation related to behavioral and psychological symptoms of dementia (BPSD) (Bosman et al., 2025; Lee et al., 2025; Prusaczyk et al., 2020; Surr et al., 2017). By reducing reliance on untrained support and improving post-discharge continuity, such a workforce has the potential to make better use of existing resources. However, broader macroeconomic effects lie beyond the scope of this study. This is particularly relevant in settings where caregivers must manage complex daily needs with limited formal assistance.

The emotional toll of caregiving was especially apparent in participants’ descriptions of exhaustion, anxiety, and burnout. These experiences reinforce the need for psychosocial support not only for PLWD, but also for family caregivers themselves. Structured psychoeducational interventions, including counseling and telephone-based support, have been shown to reduce burden and distress (Ahmad et al., 2024; Lin et al., 2025; Matsah et al., 2022; Murali et al., 2025; Tay et al., 2025; Thai et al., 2024). However, Malaysia’s mental health workforce remains limited, with only 410 registered psychologists serving a population of 35 million, far below World Health Organization recommendations (Hamzah & Othman, 2024; Ng et al., 2018; Salwana et al., 2025; Shields et al., 2017). Pragmatic workforce measures, such as rapid upskilling, recruitment, and the deployment of additional psychologists and therapists, are therefore needed to support both the national dementia policy and caregiver well-being.

More broadly, recent international reviews emphasize that direct care workers constitute the backbone of dementia care but remain poorly studied regarding training, career pathways, and retention (Travers Altizer et al., 2025). Our findings echo these concerns in a Malaysian setting, where caregivers repeatedly reported a lack of trained personnel to provide basic home-based support, psychoeducation, and service navigation. By calling for updated NOSS and DESCUM standards, we extend this global conversation to the middle-income ASEAN context and identify practical levers to strengthen para-professional career pathways.

As part of a person-centered dementia care model, psychological, physical, and occupational therapies should be integrated more closely with geriatric clinic services to reduce fragmentation, minimize repeated appointments, and support meaningful engagement for both persons living with dementia and caregivers (Ashbourne et al., 2021; Prusaczyk et al., 2020; Surr et al., 2017). Such services may be delivered individually or in groups, but should also be coordinated around caregiver needs (Weissberg, 2020). These kinds of seamless referral systems are associated with better quality of life, fewer hospitalizations, and reduced caregiver burnout (Prince et al., 2016; Surr et al., 2017).

## Limitations

This study has several limitations that should be considered when interpreting the findings. First, data were collected from a single public healthcare site, which may limit the transferability of findings to other settings, particularly private or rural care contexts with differing resource availability. This is especially relevant because dementia care pathways, workforce density, and access to community support may vary substantially across Malaysian regions and between public and private sectors. Second, the study focused exclusively on family caregivers and did not include the perspectives of persons living with dementia (PLWD), thereby capturing caregiving experiences from a single viewpoint and potentially omitting important insights into care needs and service interactions. Although this approach was appropriate for the present caregiver-centered design, it means that dyadic experiences and the lived perspective of PLWD could not be examined directly. Third, the interim cutoff at 67 participants, driven by conference timelines, may have constrained further exploration of less common or divergent caregiving experiences; however, observed thematic redundancy across core domains suggests that the principal patterns of burden and unmet needs were adequately captured. This should be interpreted as adequate for thematic coverage rather than as evidence of statistical representativeness. Finally, because the sample was heavily skewed toward female family caregivers, our findings may under-represent the perspectives and coping strategies of male caregivers. This gendered composition is consistent with caregiving norms in Malaysia, but it still limits the extent to which the findings reflect the full diversity of caregiver experiences.

## Conclusion

The profound challenges reported by dementia caregivers in Malaysia underscore a critical systemic gap: the lack of a trained para-professional workforce to provide essential transitional and community-based support. This study demonstrates that effective dementia care requires a multidisciplinary team, yet the current system, as defined by outdated occupational standards (Institute of Public Health, 2019; Department of Skills Development, n.d.), leaves families to cope on their own. Taken together with the emotional, informational, and economic strain documented in the Results, these findings show that caregiver burden is not simply a private family issue but a system-level care gap. To operationalize national policies like the *Pelan Tindakan Perkhidmatan Kesihatan Warga Emas 2023–2030* and the *Dementia Action Plan 2023–2030*, an urgent expansion of the NOSS is imperative. Introducing standardized para-professional roles (NOSS Levels 1–3) would create a vital bridge between clinics and homes, ensuring continuous, coordinated care for persons living with dementia who cannot navigate their own needs (D’Silva, 2025; Goodson et al., 2021). At minimum, such roles could support caregiver education, welfare navigation, post-discharge follow-up, and basic psychosocial support, thereby translating policy into practical assistance at the household level. Strategic investment in this workforce is the essential next step to translate policy into practice, directly improving the well-being of patients and caregivers alike. More broadly, the study highlights the need for dementia policy to move beyond clinical service expansion alone and toward integrated care systems that recognize and support the family caregivers who sustain care at home.

## Data Availability

Due to privacy and confidentiality considerations, the data are not publicly available.*

## Appendix

### Appendix: Qualitative Caregiver Narratives by Thematic Domain

#### Domain 1: Emotional Impact and Coping (Q2)

• **Theme: Emotional Exhaustion and Burnout**
  o “Emotionally tired, depressed, no life of my own. No coping strategies.” (Caregiver 9, Daughter)
  o “Angry and hopeless. No coping strategy as there is no time to manage my own stress.” (Caregiver 18, Daughter)
• **Theme: Intrafamilial Strain and Identity Conflict**
  o “Heartbreaking and painful when husband’s siblings accused [me] of being a bad wife... Husband complains to others that wife [I am] is his boss and is a slave driver who abuses him.” (Caregiver 54, Wife)
• **Theme: Emotional Numbness as Adaptation**
  o “[Previous caregiving experience] helped me to become acceptable. Now, I have become numb and emotionless.” (Caregiver 67, Wife)

#### Domain 2: Economic Burden (Q5a–d)

• **Theme: Depletion of Savings and Retirement Funds**
  o “[I am] using [my] EPF and interest from Fixed Deposits to pay for expenses. I need people to help me.” (Caregiver 57, Wife)
• **Theme: High Cost of Recurring Consumables**
  o “I am a pensioner. So far manageable. But goods are expensive now. Diapers are RM700 per month.” (Caregiver 23, Wife)
• **Theme: Occupational Loss and Dependency**
  o “As mom declines, I need to stop work and financially depend on others, which I don’t like.” (Caregiver 59, Daughter)

#### Domain 3: Information and Communication (Q3)

• **Theme: Lack of Professional Context for Dementia**
  o “ED [Emergency Department] staff don’t understand my father is dementia patient. They keep saying wait, [even though] my father was restless.” (Caregiver 14, Daughter)
• **Theme: Ambiguity Regarding Disease Progression**
  o “Still vague about progression of illness and how to prepare mentally, physically and financially.” (Caregiver 21, Son)
• **Theme: Communication Barriers and Medical Jargon**
  o “Miscommunication with healthcare professionals—medical terms, doctors cannot fully understand caregiver’s struggles, doctors only say [one] needs to be patient.” (Caregiver 65, Daughter)

#### Domain 4: Social Support and Lifestyle Impact (Q1 & Q4)

• **Theme: Social Isolation and Deprivation**
  o “My needs are being deprived. Social life affected. I have no friends and unable to work.” (Caregiver 1, Wife)
• **Theme: Living in “Survival Mode”**
  o “Yes, on survival mode.” (Caregiver 47, Daughter)
• **Theme: Feeling “Trapped” without a Break**
  o “Angry as everything is all on me. No way out and feel trapped.” (Caregiver 8, Wife)
  o “No personal life. She gives 100% of her life to taking care of her mother.” (Caregiver 5, Daughter)
